# A graph neural network framework based on preference-aware graph diffusion for recommendation

**DOI:** 10.3389/fpsyt.2022.1012980

**Published:** 2022-10-13

**Authors:** Tao Shu, Lei Shi, Chuangying Zhu, Xia Liu

**Affiliations:** ^1^Information Technology Center, Sichuan Vocational and Technical College, Suining, China; ^2^State Key Laboratory of Media Convergence and Communication, Communication University of China, Beijing, China; ^3^Guangxi Key Laboratory of Trusted Software, Guilin University of Electronic Technology, Guilin, China; ^4^School of Physics and Electronic Information, Yantai University, Yantai, China

**Keywords:** point-of-interest recommendation, user preference, graph convolutional network, temporal context, spatial context

## Abstract

Transforming user check-in data into graph structure data is a popular and powerful way to analyze users' behaviors in the field of recommendation. Graph-based deep learning methods such as graph embeddings and graph neural networks have shown promising performance on the task of point-of-interest recommendation in recent years. Despite effectiveness, existing methods fail to capture deep graph structural information, leading the suboptimal representations. In addition, they lack the ability of learning the influences of both global preference and user preference on the check-in behavior. To address the aforementioned issues, we propose a general framework based on preference-aware graph diffusion, named PGD. We first construct two types of graphs to represent the global preference and user preference. Then, we apply a graph diffusion process to capture the structural information of the generated graphs, resulting in weighted adjacency matrices. Finally, graph neural network-based backbones are introduced to learn the representations of users and POIs on weighted adjacency matrices. A learnable aggregation module is developed to learn the final representations from global preference and user preference adaptively. Extensive experiments on four real-world datasets demonstrate the superiority of PGD on POI recommendation, compared with the mainstream graph-based deep learning methods.

## Introduction

Location-based social networks (LBSNs) have attracted a large number of users to share their experience on the Internet in recent years. For example, users may submit comments about a restaurant when they visit that place in Yelp, a famous location-based social network (1, 2). That restaurant is called point-of-interest (POI), which means a place that attracts a user's interest. As the scale of LBSNs increases, more and more users tend to record their activities on the **p**latform, accumulating enormous check-in data. Such large amount of data offers the opportunities to provide the personalized recommendation service for users when they do not know where to go. This recommendation service is called POI recommendation, which has been a popular service of an LBSN over the past decade.

In the field of POI recommendation, the activities of users are recorded as check-in records, which are usually represented by graph structured data. Thus, graph-based deep learning methods are popular and powerful tools to capture the user preference in this application scenario. A general idea of graph-based deep learning (3) methods is to transform the check-in records into a variety of graphs, such as the user–timestamp graph, to model the user preference from various perspectives of factors (e.g., temporal influence). Graph embeddings (4, 5) are typical methods for learning the representations of users and POIs. These methods use the techniques of graph representation learnings, such as Deepwalk (6) and LINE (7), to learn the latent representations of nodes in the generated graphs.

Despite effectiveness, existing methods share two common weaknesses:

1. Hard to capture the deep structural relations of POIs from the generated graph. Existing methods mostly apply or design graph representation methods on the original generated graphs. Although effective for learning node representations, the generated graphs only hold on the relevance of POIs and their immediate neighbors, hard to preserve deep structural relations. Classical graph embeddings (6–8) only pay attention to a limited range of neighbors. Even though stacking several graph neural networks can relieve this impact, the over-smoothing problem (9–12) of graph neural networks will also lead to suboptimal representation.
2. Unable to learn presentations of users from global and personalized preferences. Graphs are constructed using check-in records of all users in most graph-based deep learning methods. Such graphs only preserve the global preference, ignoring the personalized preference of a unique user (13, 14). This drawback could affect the model performance for personalized recommendation.

To address the aforementioned issues, we propose a general graph neural network framework for POI recommendation based on preference-aware graph diffusion, named PGD. We first construct two types of graphs to preserve global and personalized preferences, respectively. Then, we conduct the graph diffusion process on generated graphs to capture deep graph structural information, which resulted in a series of weighted matrices. Finally, a graph neural network-based backbone is applied to learn the representations of POIs according to the weighted matrices. We propose a learnable aggregation module to learn the user preference from both global and personalized aspects. We conduct extensive experiments on three widely used datasets from real-world LBSNs. The experimental results have demonstrated the superiority of PGD, compared with existing graph-based deep learning methods. The contributions of this article are as follows:

- We propose PGD, a general framework, for POI recommendation. The choice of a graph neural network as the backbone is arbitrary.
- We conduct the graph diffusion process to capture deep structural information, which is neglected in most existing methods.
- We propose a learnable aggregation module to learn the user preference from both global and personalized aspects adaptively.
- We conduct extensive experiments on real-world datasets to validate effectiveness of the method. The results show that our proposed PGD outperforms existing graph-based deep learning methods.

The rest of the article is organized as follows: In Section Related work, we briefly review the related works on graph-based deep learning methods for POI recommendation. In Section Preliminaries, we provide some key definitions of terms used in this article, including the definitions of graphs and LBSNs. In Section Proposed framework, we detail our proposed method, including the key designs and learning methods of model parameters. In Section Experiments, we introduce the settings of experiments and report the results. Finally, we conclude this article and outline the future directions in Section Conclusion.

## Related work

In this section, we review graph-based deep learning methods for the task of POI recommendation. The goal of graph-based deep learning methods, including graph embeddings and graph neural networks, is to learn the low-dimensional representation feature vectors of users and POIs from the graph-structured data generated by the check-in records of users. Then, the representation vectors are used to calculate the rank scores of all unobserved user–POI pairs. Finally, the recommendation list is created according to the rank scores from high to low.

GeoMF (15) utilizes the geography of POIs to construct the potential regions to learn the influence of POI locations on user preference. Then, a learning method based on matrix decomposition is developed to learn the representation vectors of users and POIs. POI2Vec (16) leverages the rank-based embedding method to incorporate both the geographical influence and sequential transition influence. Geo-PFM (17) conducts the Poisson distribution to capture the user mobility behaviors and takes various factors into the model for learning user preferences precisely. GE (5) is one of the typical embedding-based methods for POI recommendation. GE first transforms the check-in records into four graphs to capture the features from the aspects of geography, time, check-in pattern, and semantics. Then, a joint training method is proposed to learn the representations from the aforementioned impact factors. STA (4) defines the spatiotemporal context, which combines the location and timestamp of check-in records. Such a novel definition of the context makes it possible to capture the characteristics of users' check-in behaviors carefully. Based on this, STA utilizes the knowledge graph embedding method (18) to model the user preference through the translation-based methods. Zhang et al. (19) considered the category translation of check-in records and proposed a model named HCT to capture the dynamic preference of users according to the POIs and their categories. JLGE (20) uses a three-step strategy to learn the representation of users and POIs: First, JLGE constructs a series of graphs to represent the interactions of between users and various influence factors, such as temporal factors. Then, a graph embedding-based module (7) is applied to learn the representations of nodes. Finally, a ranking score function is used to calculate the scores of users and POIs according to the learned representation vectors. Xiong et al. (21) introduced the graph embeddings to jointly learn the representation vectors for different graphs to preserve the dynamic preference of users.

Despite their effectiveness, embedding-based methods are weak to learn more useful structural information from the check-in graphs. Thanks to the amazing ability of graph neural networks (GNNs) for learning the powerful representation from the graph-structured data, many related works have been proposed to introduce GNNs into the POI recommendation models in recent years. Wang et al. (22) utilized the GNNs to learn long- and short-term preferences of users according to the check-in graphs. Xu et al. (23) utilized the graph attention network (24) to learn the user preference from the POI and ROI levels. GGLR (25) leverages the graph neural network to learn the representations of POIs according to the newly defined two types of geographical influences: ingoing and outgoing influences. STP-UDGAT (26) develops a masked self-attention option based on the original graph attention network to exploit personalized user preferences. Zhang et al. (27) combined GNNs and long short-term memory (Bi-LSTM) to learn the user preference from the users' sequential check-in behavior, involving geographical and temporal features. For more related works, we refer to the survey (28) about deep learning-based models for POI recommendation.

The aforementioned graph-based deep learning methods are conducted on the interaction networks generated by the check-in records. However, they ignore the deep structural information on such graph-structured data, causing them to learn the suboptimal representations of users and POIs. Our proposed framework PGD utilizes the graph diffusion process to preserve the structural information of the generated graphs, further improving the effectiveness of graph-based deep learning methods.

## Preliminaries

### Definitions in LBSN

Suppose there are two sets *U* = {*u*_1_, ..., *u*_*m*_} and *P* = {*p*_1_, ..., *p*_*n*_} representing users and POIs in an LBSN. A POI *p*_*i*_ is associated with longitude and latitude coordinates, denoted *l*_*p*_*i*__. Then, we have the following definitions:

**Definition 1 (Check-in record):** Check-in records *D*_*u*_ are denoted by a tuple (*u, p, l, t*) that represents the check-in behavior of the user *u* who visited the POI *p* at the time *t* in the location *l*.

**Definition 2 (User–POI graph):** The user–POI graph *G*_*up*_ = (*V*_*up*_, *E*_*up*_) is a bipartite graph whose node set consists of two disjoint parts *V*_*up*_ = *U*+*P*. *E*_*up*_ denotes the edge set. If the user *u* visited the POI *p*, there will be an edge between nodes *u* and *p*, reflecting users' check-in records.

**Definition 3 (Global activity graph):** The global activity graph *G*_*ga*_ = (*V*_*ga*_, *E*_*ga*_) is a POI-POI interaction graph, where *V*_*ga*_ = *P*. If a user first visits the POI*p*_*i*_ and then visits *p*_*j*_ within a time frame Δ*t*, there will be an edge between nodes *p*_*i*_ and *p*_*j*_. *G*_*ga*_ is a weighted graph that describes the check-in pattern of all users. The higher the frequency of *p*_*i*_ and *p*_*j*_, the greater the weight of the edge *e*_*p*_*i*_*p*_*j*__.

**Definition 4 (Personalized activity graph):** The personalized activity graph *G*_*pa*_ = (*V*_*pa*_, *E*_*pa*_) is similar to *G*_*ga*_. The difference between them is that *G*_*pa*_ is changed for each user, describing the check-in pattern of a unique user.

### POI recommendation

Given the check-in records, the location *l*, and the timestamp *t*, the task of POI recommendation is generating a list of POIs {*p*_1_, ..., *p*_*k*_} for a user *u*, where *k* is the length of the recommendation list. These recommended POIs do not appear in the history check-in records of the user *u*.

## Proposed framework

In this section, we detail our proposed PGD. It consists of three stages: (1) generating the weight matrices based on graph diffusion, (2) learning the representations of users and POIs, and (3) optimizing the parameters.

### Graph diffusion operation

Most of the existing graph-based deep learning methods only utilize the information of immediate neighbors on the graphs generated by check-in records of users. For example, graph embeddings sample the node sequence based on the link relations between nodes. GNNs aggregate the information according to the adjacency matrix. Information from limited neighbors will lead to the suboptimal representations.

To address this problem and capture the graph structural information deeply, we conduct a graph diffusion operation on the generated graphs. We first produce a global activity graph to hold the global preference based on the definition in Section Definitions in LBSN. We produce a series of personalized activity graphs for users based on their unique check-in records to preserve the user preference according the definition in Section Definitions in LBSN.

Then, we define the graph diffusion process. Given a graph *G* and its corresponding adjacency matrix *A*, a generalized graph diffusion (29) operation is defined as (30, 31) as follows:


(1) $$\begin{array}{l} Diff\left(G\right)=\overset{\infty }{\underset{x=0}{\sum }}\theta_{x}T^{x}. \end{array}$$

where *T* is the transition matrix, produced by a normalized version of *A*, that is, symmetric normalization. Equation (1) is a general form. In practice, we apply the personalized PageRank to conduct the diffusion process by setting $\theta_{x}\text{=}\alpha {\left(1-\alpha \right)}^{x}$, where α ∈ (0, 1) denotes the teleport probability. Let *S* denote the result of *Diff*(*G*) and *S* a weighted graph, where the weight of an edge describes the structural information bias between two nodes on the graph. The large weight represents the strong topology similarity so that *S* preserves deeply structural information compared with the original adjacency matrix.

The motivation to conducting the diffusion operation is that the result of the diffusion process provides a more precise description of the similarity between two nodes, which is beneficial for learning the representations of users and POIs from the generated graphs based on check-in records. For each user, the adjacency matrix of the personalized activity graph is different from that of other users so that the resultant matrix of the diffusion matrix is also different, thus preserving the personalized preference of users.

### GNN-based backbone

After the graph diffusion process, we obtain the weighted matrices of the generated graphs, $S^{G_{ga}}$ from the global activity graph *G*_*ga*_ and $S^{G_{pa}}$ from the personalized activity graph *G*_*ga*_. We use the row normalization method to normalize them since we only consider the relations of the central node and their neighbors. We further learn the representations of users and POIs based on the aforementioned matrices through a GNN-based backbone.

For a user *u*, we have two matrices, $S^{G_{ga}}$ and $S^{{G^{{}_{u}}}_{pa}}$. These matrices preserve the relations of POIs from global and personalized perspectives. For learning the representation of POIs, we apply GNNs on the aforementioned matrices. It is noteworthy that the selection of GNNs is arbitrary, demonstrating the flexibility of our proposed method. In this article, we use two GNNs, GCN and GAT, to learn the representations of users and POIs. The GCN and GAT are popular and powerful GNNs for learning the node representations of graphs. Note that our proposed PGD is a general framework, and most GNNs could be introduced into PGD for POI recommendation.

**GCN** (32): The GCN is a typical GNN that utilizes the first-order Laplace smoothing for aggregating the information from neighbors. A GCN layer is defined as follows:


(2) $$\begin{array}{l} {\text{H}}^{\left(l+1\right)}=\sigma \left(\text{S}{\text{H}}^{\left(l\right)}{\text{W}}^{\left(l\right)}\right), \end{array}$$

where *H* denotes the representations of POIs and *W* denotes the learnable parameter matrix. Since there are no raw features for POIs, we randomly use a matrix as the input of the first layer of the GCN.

**GAT** (24): Different from the GCN that aggregates information based on the node degree, the GAT introduces the attention layer to guide the aggregation process. A GAT layer is defined as follows:


(3) $$\begin{array}{l} {\text{H}}^{\left(l+1\right)}=\sigma \left(\left(\text{S}⊙\text{M}\right){\text{H}}^{\left(l\right)}{\text{W}}^{\left(l\right)}\right), \end{array}$$

where *M* is the attention matrix of node pairs and ⊙ denotes the element-wise multiplication. We modify the original GAT layer to introduce the diffusion matrix into the aggregation of the GAT.

After the GNN backbone, we obtain the representation of POIs from the global graph ${\text{S}}^{G_{ga}}$and personalized graph ${\text{S}}^{{G^{{}_{u}}}_{pa}}$, denoted as ${\text{H}}^{G_{ga}}$ and ${\text{H}}^{{G^{u}}_{pa}}$, respectively. We use ${\text{H}}^{G_{ga}}$ as the final representations *P* for POIs for the reason that the global graph contains more information than the personalized graph.

For calculating the representations of users, we define a learnable aggregation module to learn the final representations. Suppose the visited list of POIs in the check-in records of the user *u* is *C* = {*p*_1_, ..., *p*_*c*_}, we develop the following strategy to learn the representation *U*:


(4) $$\begin{array}{l} {\text{U}}_{u}=\frac{1}{\left|C\right|}\underset{p\in C}{\sum }LA\left({\text{H}}^{G_{ga}},{\text{H}}^{{G^{u}}_{pa}}\right), \end{array}$$

(5) $$\begin{array}{l} LA\left(H^{G_{ga}},H^{{G^{u}}_{pa}}\right)=\beta_{ga}\cdot {\text{H}}^{G_{ga}}+\beta_{pa}\cdot {\text{H}}^{{G^{u}}_{pa}}. \end{array}$$

where *LA*(·) denotes the learnable aggregation module, and β_*ga*_ and β_*pa*_ are learnable scalars for calculating the representations of users adaptively. We further use the SoftMax function to guarantee the values of β_*ga*_ and β_*pa*_ are in the reasonable range.

Intuitively, the representation of a user comes from the global preference and personalized preference. The function *LA*(·) is capable of preserving the preferences from the previous two aspects by introducing the learnable aggregation factors.

### Parameter optimization

To learn the parameters of the proposed model, we adopt the general optimization framework, Bayesian personalized ranking (33), for its wide usage in the field of recommendation (13, 14, 34). The objective function of proposed method is defined as follows:


(6) $$\begin{array}{l} L\text{=-}\overset{m}{\underset{u=1}{\sum }}\underset{p_{i}\in D_{u}}{\sum }\underset{p_{j}\notin D_{u}}{\sum }\text{In}\varphi \left({\text{U}}_{u}\cdot {{\text{P}}^{\tau }}_{p_{i}}-{\text{U}}_{u}\cdot {{\text{P}}^{\tau }}_{p_{j}}\right)+\zeta ||\Theta |{|}^{2} \end{array}$$

where φ(·) denotes the sigmoid function, ζ denotes the regularization coefficient, and Θ denotes the parameters of PGD. By minimizing Equation (6) with the stochastic gradient descent algorithm, we can learn the representations for users and POIs.

## Experiments

In this section, we introduce the experiments conducted in this article. We first introduce the experimental settings, including datasets, evaluation metrics, and baselines. Then, we report the results of experiments and provide related analyses.

### Datasets

We use three popular real-world datasets, namely, Yelp (27), Foursquare (27) and Gowalla (27), for experiments in this article. These three datasets are collected from the famous LBSNs: Yelp, Foursquare, and Gowalla, respectively. For each dataset, we perform the data cleaning process and produce the check-in records, obeying the format described in Section Definitions in LBSN. In addition, we remove the users whose check-in records are < 20. We also remove the POIs whose visitors are < 20. The statistics of datasets are reported in Table 1.

**Table 1:** Statistics of datasets.

**Dataset**	**Users**	**POIs**	**Check-in records**
Yelp	24,655	15,213	689,410
Foursquare	21,037	21,408	828,132
Gowalla	14,654	26,794	962,013

We split each dataset into three sets according to the check-in timestamp: the former 60% is the train set, the latest 20% is the test set, and the remaining 20% is the validation set.

### Evaluation metrics

In this article, we choose the widely used evaluation metrics, precision (27) and recall (35), to measure the recommendation performance of all models:


(7) $$\begin{array}{l} Precision=\frac{\left|D_{test}\cap Top\text{\_}k\right|}{\left|Top\text{\_}k\right|} \end{array}$$

(8) $$\begin{array}{l} Recall=\frac{\left|D_{test}\cap Top\text{\_}k\right|}{\left|D_{test}\right|}, \end{array}$$

where *D*_*test*_ denotes the test set and *Top*_*k* denotes the recommendation list of POIs. We set the length of the list to 10 for experiments. Precision denotes the ratio of successfully recommended POIs in the recommendation list. Recall denotes the ratio of the ratio of successfully recommended POIs in all unvisited POIs.

### Baselines

In this article, we select the following methods as the baselines for experiments:

GeoMF (15): GeoMF utilizes the latent factor model to capture the influence of geographical factors on the check-in behavior of users.

Geo-PFM (17): The geographical probabilistic factor model adopted Poisson distribution can effectively model the user mobility patterns by capturing the geographical influences.

POI2Vec (16): POI2Vec is a ranking-based model that utilizes the sequential influence of check-in records and jointly learns the preference of POIs and sequential transition.

GE (5): GE is a generic graph-based embedding model, which jointly captures the sequential effect, geographical influence, temporal cyclic effect, and semantic effect in a unified way.

STA (4) STA introduces the translation-based model to capture the spatiotemporal context for learning the check-in pattern of users.

For the proposed method PGD, we provide two variants implemented by GCN and GAT, namely, PGD-GCN and PGD-GAT, respectively.

For baselines, we use the recommended settings of the hyper-parameters from previous studies. For PGD, we use the grid search method to find the suitable values of the coefficient ζ of the regularization in Equation (6) and the learning rate *lr* of the optimizer. The research spaces are ζ ∈ {0.005, 0.001, 0.0005} and *lr* ∈ {0.01, 0.005, 0.001}. In this article, we set ζ = 0.0005 and *lr* = 0.001 for experiments.

### Impact of time threshold

In this section, we study the influence of the time threshold Δ*t*, determining the construction of *G*_*ga*_ and *G*_*pa*_.

**Figure 1:**
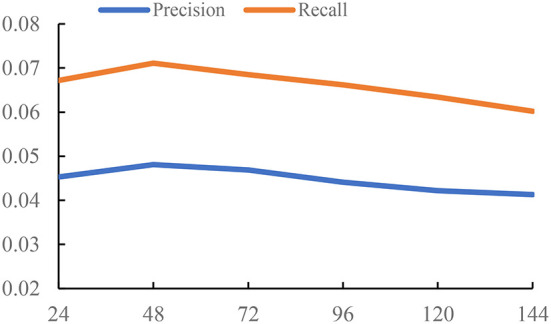
Impact of time threshold on Yelp.

**Figure 2:**
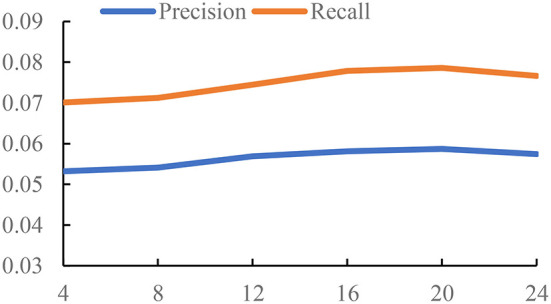
The impact of time threshold on Foursquare.

**Figure 3:**
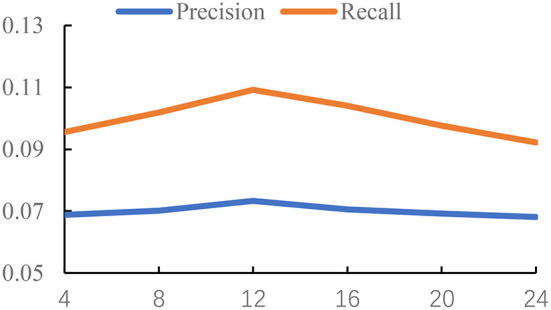
Impact of time threshold on Gowalla.

The time threshold Δ*t* controls the density of the graph. If we set a small value Δ*t*, we will get a relatively sparse graph, which means there are less interactions between POIs. Also, it is hard to learn the meaningful representations on a sparse graph. But if we set a large value Δ*t*, the edges in the constructed graph are unable to accurately capture the relations of POIs from the check-in pattern of users. Thus, we conduct the experiments to study the influence of the time threshold Δ*t*. The settings of three datasets are different due to the different check-in data. For Foursquare and Gowalla, we set Δ*t* from {4, 8, ..., 24}. For Yelp, we set Δ*t* from {24, 48, ..., 144}. The unit of Δ*t* is hour; the reason is that Yelp is a reviewer dataset, and the check-in time is recorded by day. Foursquare and Gowalla are the real-time check-in datasets; thus, we have more information on the check-in time on these datasets. So, the value of Δ*t* on Foursquare and Gowalla is smaller than that on Yelp. We use the GCN as the backbone of experiments. The results are reported in Figures 1–3.

From the results of Figures 1–3, we can observe that the time threshold makes a great influence on the model performance. The reason is that the time threshold determines the quality of the generated graphs. A suitable time threshold is beneficial to construct a graph with high quality to describe the relations of POIs, further improving the model performance. We can also observe that the value of achieving the best performance is sensitive to the datasets. This is because different datasets exhibit different check-in patterns of users. Based on the results, we set Δ*t* to 48 on Yelp. For Foursquare, we set it to 16. For Gowalla, we set it to 12.

### Comparison of methods

We run all methods on three datasets with 10 random seeds and report the average of all evaluation metrics. The results are summarized in Tables 2–4.

**Table 2:** Results of all methods on Yelp.

**Method**	**Precision**	**Recall**
GeoMF	0.0223	0.0327
Geo-PFM	0.0281	0.0415
POI2Vec	0.0354	0.0514
GE	0.0432	0.0643
STA	0.0439	0.0652
PGD-GCN	0.0481	0.0711
PGD-GAT	**0.0509**	**0.0754**

**Table 3:** Results of all methods on Foursquare.

**Method**	**Precision**	**Recall**
GeoMF	0.0319	0.0423
Geo-PFM	0.0322	0.0441
POI2Vec	0.0373	0.0536
GE	0.0491	0.0693
STA	0.0521	0.0732
PGD-GCN	0.0587	0.0786
PGD-GAT	0.0595	0.0798

**Table 4:** Results of all methods on Gowalla.

**Method**	**Precision**	**Recall**
GeoMF	0.0459	0.0636
Geo-PFM	0.0471	0.0678
POI2Vec	0.0644	0.0916
GE	0.0693	0.0983
STA	0.0702	0.0994
PGD-GCN	0.0733	0.1092
PGD-GAT	0.0745	0.1125

From Tables 2–4, we can observe that our proposed methods PGD-GCN and PGD-GAT consistently outperform other baselines, demonstrating the superiority of the proposed framework. In addition, PGD-GAT outperforms PGD-GCN, which indicates that introducing the attention mechanism benefits learning the representation vectors of users and POIs. Considering the best results of three datasets, the lowest one is from Yelp. This is because the dataset of Yelp is most sparse, compared with Foursquare and Gowalla. This phenomenon also implies that the data sparsity has a great influence on the performance of the POI recommendation task.

### Ablation study

In this section, we first design ablation studies to measure the contribution of the proposed learnable aggregation module to the model performance. We also use the GCN as the backbone model. We propose two variants, PGD-GCN-0 and PGD-GCN-1. PGD-GCN-0 denotes that only the global preference is considered in the model. PGD-GCN-1 means that the learnable aggregation is removed. The results are reported in Tables 5–7.

**Table 5:** Results of variants on Yelp.

**Method**	**Precision**	**Recall**
PGD-GCN-0	0.0442	0.0661
PGD-GCN-1	0.0451	0.0675
PGD-GCN	0.0481	0.0711

**Table 6:** Results of variants on Foursquare.

**Method**	**Precision**	**Recall**
PGD-GCN-0	0.0512	0.0709
PGD-GCN-1	0.0541	0.0755
PGD-GCN	0.0587	0.0786

**Table 7:** Results of variants on Gowalla.

**Method**	**Precision**	**Recall**
PGD-GCN-0	0.0708	0.0998
PGD-GCN-1	0.0721	0.1054
PGD-GCN	0.0733	0.1092

The results of Tables 5–7 have demonstrated that our proposed learnable aggregation module is helpful to learn the precise representations of users.

Then, we design experiments to validate the effectiveness of the graph diffusion process. As mentioned before, the diffusion process is helpful to capture the deep graph structural information and further promote to learn the relations of POIs. We consider a variant of PGD where the graph diffusion process is removed, PGD-GCN-RW. The GCN backbone is also applied in experiments. The results are reported in Tables 8–10.

**Table 8:** Results of variants on Yelp.

**Method**	**Precision**	**Recall**
PGD-GCN-RW	0.0436	0.0648
PGD-GCN	0.0481	0.0711

**Table 9:** Results of variants on Foursquare.

**Method**	**Precision**	**Recall**
PGD-GCN-RW	0.0498	0.0699
PGD-GCN	0.0587	0.0786

**Table 10:** Results of variants on Gowalla.

**Method**	**Precision**	**Recall**
PGD-GCN-RW	0.0703	0.0996
PGD-GCN	0.0733	0.1092

The results from Tables 8–10 have proved that the graph diffusion process is necessary to learn the powerful representations of users and POIs. With the graph diffusion process, the performance of the model has been significantly improved.

### Discussion of results

In the experiments, we first study the influence of the settings of the time threshold. The results show that a suitable value of the time threshold can help model improve the recommendation effectiveness. Then, we compare our proposed PGD with baselines on real-world datasets. The results indicate the superiority of PGD for the POI recommendation task. Finally, we conduct ablation studies to explore the gain of key designs of PGD, that is, graph diffusion and learnable aggregation module. The results show that all key designs are beneficial for improving the model performance.

## Conclusion

In this article, we propose a general GNN-based framework, named PGD. PGD first constructs two types of graphs to preserve the global and personalized preferences. Then, a graph diffusion process is applied to capture the deep graph structural information. Finally, a GNN-based backbone is developed to learn the representations of POIs. For the representations of users, we propose a learnable aggregation module to learn the features from both global and personalized aspects adaptively. We conduct extensive experiments on three real-world datasets. The experimental results show that our proposed method outperforms the mainstream POI recommendation methods.

PGD is a general framework, and it can utilize most GNNs to learn the representations of users and POIs and show its high flexibility. The superiority of PGD demonstrates that the graph diffusion process is beneficial for learning the powerful representations, which reveals that leveraging high-order structural relations is a crucial point for improving the model performance.

For the future directions, although PGD utilizes the graph diffusion process to preserve the structural information, it relies on the rich check-in records of users. It is hard to capture the relations of unobserved POIs based on the graph diffusion so that we plan to introduce various similarity-based techniques to estimate the semantic relevance between all POIs. Such pre-computed similarities are helpful to relieve the impact of data sparsity.

## Data availability statement

All datasets can be downloaded from the following websites: https://www.yelp.com/dataset (for Yelp), https://sites.google.com/site/yangdingqi/home/foursquare-dataset (for Foursquare), http://snap.stanford.edu/data/loc-gowalla.html (for Gowalla).

## Author contributions

TS and LS designed the overall framework and conceived the idea of this paper. TS analyzed the data using correlation algorithms. TS and CZ wrote the paper. XL helped in typesetting and revising the paper, and modified the English grammar. All authors contributed to the article and approved the submitted version.

## Funding

This work was supported by the Fundamental Research Funds for the Central Universities (No. CUC220C011, CUC22GZ038) and Youth Fund Project of Guangxi Natural Science Foundation (No. 2021GXNSFBA196054).

## Conflict of interest

The authors declare that the research was conducted in the absence of any commercial or financial relationships that could be construed as a potential conflict of interest.

## Publisher's note

All claims expressed in this article are solely those of the authors and do not necessarily represent those of their affiliated organizations, or those of the publisher, the editors and the reviewers. Any product that may be evaluated in this article, or claim that may be made by its manufacturer, is not guaranteed or endorsed by the publisher.

## References

[1] Shi L Du J Cheng G Liu X Xiong Z Luo J. Cross-media search method based on complementary attention and generative adversarial network for social networks. Int J Intell Syst. (2022) 37:4393–416. 10.1002/int.22723

[2] Shi L Song G Cheng G Liu X. A user-based aggregation topic model for understanding user's preference and intention in social network. Neurocomputing. (2020) 413:1–13. 10.1016/j.neucom.2020.06.099

[3] Zhang Y Yu L Fang Z Xiong NN Zhang L Tian H. An end-to-end deep learning model for robust smooth filtering identification. Fut Gen Comput Syst. (2022) 127:263–75. 10.1016/j.future.2021.09.004

[4] Qian T Liu B Nguyen QVH Yin H. Spatiotemporal representation learning for translation-based POI recommendation. ACM Trans Infm Syst. (2019) 37:1–24. 10.1145/3295499

[5] Xie M Yin H Wang H Xu F Chen W Wang S. Learning graph-based poi embedding for location-based recommendation. In: Proceedings of the 25th ACM International on Conference on Information and Knowledge Management. Indianapolis, IN: Association for Computing Machinery (2016). p. 15–24. 10.1145/2983323.2983711

[6] Perozzi B Al-Rfou R Skiena S. Deepwalk: online learning of social representations. In: Proceedings of the 20th ACM SIGKDD International Conference on Knowledge Discovery and Data Mining. New York, NY: Association for Computing Machinery (2014). p. 701–10. 10.1145/2623330.2623732

[7] Tang J Qu M Wang M Zhang M Yan J Mei Q. Line: large-scale information network embedding. In: Proceedings of the 24th International Conference on World Wide Web. Florence: International World Wide Web Conferences Steering Committee (2015). p. 1067–77. 10.1145/2736277.2741093

[8] Grover A Leskovec J. node2vec: scalable feature learning for networks. In: Proceedings of the 22nd ACM SIGKDD International Conference on Knowledge Discovery and Data Mining. (2016). p. 855–64. 10.1145/2939672.2939754 27853626 PMC5108654

[9] Li Q Han Z Wu XM. Deeper insights into graph convolutional networks for semi-supervised learning. In: Thirty-Second AAAI Conference on Artificial Intelligence. (2018). p. 3538–45. 10.1609/aaai.v32i1.11604

[10] Chen D Lin Y Li W Li P Zhou J Sun X. Measuring and relieving the over-smoothing problem for graph neural networks from the topological view. In: P*roceedings of the AAAI Conference on Artificial Intelligence*. (2020). Vol. 34, p. 3438–45. 10.1609/aaai.v34i04.5747

[11] Zhou K Dong Y Wang K Lee WS Hooi B Xu H. Understanding and resolving performance degradation in deep graph convolutional networks. In: Proceedings of the 30th ACM International Conference on Information and Knowledge Management. (2021). p. 2728–37. 10.1145/3459637.3482488

[12] Liu M Gao H Ji S. Towards deeper graph neural networks. In: Proceedings of the 26th ACM SIGKDD International Conference on Knowledge Discovery and Data Mining. (2020). p. 338–48. 10.1145/3394486.3403076

[13] Li X Jiang M Hong H Liao L. A time-aware personalized point-of-interest recommendation via high-order tensor factorization. ACM Trans Inform Syst. (2017) 35:1–23. 10.1145/3057283

[14] He J Li X Liao L. Category-aware next point-of-interest recommendation via listwise bayesian personalized ranking. In: Twenty-Sixth International Joint Conference on Artificial Intelligence. Melbourne, VIC: IJCAI (2017). Vol. 17, p. 1837–43. 10.24963/ijcai.2017/255

[15] Lian D Zhao C Xie X Sun G Chen E Rui Y. GeoMF: joint geographical modeling and matrix factorization for point-of-interest recommendation. In: Proceedings of the 20th ACM SIGKDD International Conference on Knowledge Discovery and Data Mining. (2014). p. 831–40. 10.1145/2623330.2623638

[16] Feng S Cong G An B Chee YM. Poi2vec: Geographical latent representation for predicting future visitors. In: Thirty-First AAAI Conference on Artificial Intelligence. (2017). p. 102–8. 10.1609/aaai.v31i1.10500

[17] Liu B Xiong H Papadimitriou S Fu Y Yao Z. A general geographical probabilistic factor model for point of interest recommendation. IEEE Trans Knowl Data Eng. (2014) 27:1167–79. 10.1109/TKDE.2014.2362525

[18] Lin Y Liu Z Sun M Liu Y Zhu X. Learning entity and relation embeddings for knowledge graph completion. In: Twenty-Ninth AAAI Conference on Artificial Intelligence. (2015). p. 2181–7. 10.1609/aaai.v29i1.9491

[19] Zhang L Sun Z Zhang J Kloeden H Klanner F. Modeling hierarchical category transition for next POI recommendation with uncertain check-ins. Inf Sci. (2020) 515:169–90. 10.1016/j.ins.2019.12.006

[20] Christoforidis G Kefalas P Papadopoulos A Manolopoulos Y. Recommendation of points-of-interest using graph embeddings. In: 2018 IEEE 5th International Conference on Data Science and Advanced Analytics. (2018). p. 31–40. 10.1109/DSAA.2018.00013

[21] Xiong X Xiong F Zhao J Qiao S Li Y Zhao Y. Dynamic discovery of favorite locations in spatio-temporal social networks. Inform Process Manag. (2020) 57:102337. 10.1016/j.ipm.2020.102337

[22] Wang D Wang X Xiang Z Yu D Deng S Xu G. Attentive sequential model based on graph neural network for next poi recommendation. World Wide Web. (2021) 24:2161–84. 10.1007/s11280-021-00961-9

[23] Xu H Wei J Yang Z Wang J. Graph attentive network for region recommendation with poi-and roi-level attention. In: Asia-Pacific Web (APWeb) and Web-Age Information Management (WAIM) Joint International Conference on Web and Big Data. (2020). p. 509–16. 10.1007/978-3-030-60259-8_37

[24] Veličković P Cucurull G Casanova A Romero A Lio P Bengio Y. Graph attention networks. arXiv preprint arXiv:1710.10903. (2017). 10.48550/arXiv.1710.10903

[25] Chang B Jang G Kim S Kang J. Learning graph-based geographical latent representation for point-of-interest recommendation. In: Proceedings of the 29th ACM International Conference on Information and Knowledge Management. (2020). p. 135–44. 10.1145/3340531.3411905

[26] Lim N Hooi B Ng SK Wang X Goh YL Weng R. STP-UDGAT: spatial-temporal-preference user dimensional graph attention network for next POI recommendation. In: Proceedings of the 29th ACM International Conference on Information and Knowledge Management. (2020). p. 845–54. 10.1145/3340531.3411876

[27] Zhang J Liu X Zhou X Chu X. Leveraging graph neural networks for point-of-interest recommendations. Neurocomputing. (2021) 462:1–13. 10.1016/j.neucom.2021.07.063

[28] Islam MA Mohammad MM Das SSS Ali ME. A survey on deep learning based Point-of-Interest (POI) recommendations. Neurocomputing. (2022) 472:306–25. 10.1016/j.neucom.2021.05.114

[29] Zhang Y Liu T Cattani C Cui Q Liu S. Diffusion-based image inpainting forensics via weighted least squares filtering enhancement. Multimed Tools Appl. (2021) 80:30725–39. 10.1007/s11042-021-10623-7

[30] Klicpera J Weißenberger S Günnemann S. Diffusion improves graph learning. arXiv preprint arXiv:1911.05485. (2019). 10.48550/arXiv.1911.05485

[31] Zhao J Dong Y Ding M Kharlamov E Tang J. Adaptive diffusion in graph neural networks. Adv Neural Inf Process Syst. (2021) 34:23321–33.

[32] Kipf TN Welling M. Semi-supervised classification with graph convolutional networks. arXiv preprint arXiv:1609.02907. (2016). 10.48550/arXiv.1609.02907

[33] Rendle S Freudenthaler C Gantner Z and Schmidt-Thieme L. BPR: bayesian personalized ranking from implicit feedback. arXiv preprint arXiv:1205.2618. (2012). 10.48550/arXiv.1205.2618

[34] Jhamb Y Fang Y. A dual-perspective latent factor model for group-aware social event recommendation. Inform Process Manag. (2017) 53:559–76. 10.1016/j.ipm.2017.01.001

[35] Ji W Meng X Zhang Y. STARec: adaptive learning with spatiotemporal and activity influence for POI recommendation. ACM Trans Inform Syst. (2021) 40:1–40. 10.1145/3485631

